# Vulnerability to Climate Change of Mangroves: Assessment from Cameroon, Central Africa

**DOI:** 10.3390/biology1030617

**Published:** 2012-11-06

**Authors:** Joanna C. Ellison, Isabella Zouh

**Affiliations:** 1 School of Geography and Environmental Studies, University of Tasmania, Locked Bag 1376, Launceston 7250, Tasmania, Australia; 2 Ministry of Environment and the Protection of Nature, MINEP, PB 320 Yaoundé, Cameroon; Email: zouhtem@yahoo.com

**Keywords:** mangroves, vulnerability, sea-level rise, shoreline change, elevation, geographic information systems, pollen analysis

## Abstract

Intertidal mangrove ecosystems are sensitive to climate change impacts, particularly to associated relative sea level rise. Human stressors and low tidal range add to vulnerability, both characteristics of the Doula Estuary, Cameroon. To investigate vulnerability, spatial techniques were combined with ground surveys to map distributions of mangrove zones, and compare with historical spatial records to quantify change over the last few decades. Low technology techniques were used to establish the tidal range and relative elevation of the mapped mangrove area. Stratigraphic coring and palaeobiological reconstruction were used to show the longer term biological history of mangroves and net sedimentation rate, and oral history surveys of local communities were used to provide evidence of recent change and identify possible causes. Results showed that the seaward edge of mangroves had over two thirds of the shoreline experienced dieback at up to 3 m per year over the last three decades, and an offshore mangrove island had suffered 89% loss. Results also showed low net sedimentation rates under seaward edge mangroves, and restricted intertidal elevation habitats of all mangroves, and *Avicennia* and *Laguncularia* in particular. To reduce vulnerability, adaptation planning can be improved by reducing the non-climate stressors on the mangrove area, particularly those resulting from human impacts. Other priorities for adaptation planning in mangrove areas that are located in such low tidal range regions are to plan inland migration areas and strategic protected areas for mangroves, and to undertake management activities that enhance accretion within the mangroves.

## 1. Introduction

Mangrove forests are the primary biological systems of sheltered, sedimentary coastlines of the tropics, where trees have evolved unique adaptations for an intertidal habitat such as aerial roots, vivipary and salt regulation. Mangrove systems play an integral role at the interface between terrestrial, freshwater and marine systems, providing protection to both terrestrial and estuarine systems from high-energy marine processes. They have an important role in protecting coasts during storm and tsunami events, both by frictional reduction of wave energy and by promoting sedimentary resilience to erosion through the root mat [1,2,3,4,5]. Studies following the 2004 tsunami found that, in some places, human deaths and loss of property were reduced by the presence of coastal vegetation shielding coastal villages [2,4,6]. Reduction of wave height and energy is influenced by the structure of the mangrove forest and the type of aerial root systems.

The forests also act to filter runoff water and so protect offshore sea grass beds and coral reefs from deposition of suspended matter discharged by rivers. One of the most important values of mangroves to people is their support of ecologically and economically important fish species [7,8,9,10,11]. The ecosystem is known to act as a nursery site for many fish and crustacean species important for both commercial and subsistence purposes [10,12], enhancing juvenile survivorship and the diversity and abundance of fish in offshore waters.

Mangrove ecosystems are a significant carbon sink in terms of forest biomass as well as organic sediment accumulation [13,14]. For centuries, mangroves have provided a wide range of products for coastal communities, such as timber and fuelwood and bioactive compounds for tanning and medicinal purposes [15,16,17].

Despite these values, many mangrove systems have become degraded and destroyed [18,19,20]. As a result of conversion to development, aquaculture or agriculture, overharvesting for timber, unsustainable fishing and other extractive uses, the worldwide mangrove area fell from over 200,000 km^2^ before 1950 to 188,000 km^2^ in 1980, and to below 150,000 km^2^ by the end of 2000 [19], with the vast majority of that loss after 1980. Asia has suffered the highest losses [21]; and because data used in these assessments preceded the 2004 Asian tsunami, the losses were due to human impacts [18,22,23]. A recent GIS reassessment using Landsat archives put the 2000 global extent of mangroves at only 137,760 km^2^ [20]. The implied rates of loss are faster than of tropical rainforests or coral reefs [23], but generally receive far less attention [19].

Climate change has begun to compound the effects of many of these threats, as reviewed in the next section. Degradation and loss of these coastal systems due to climate change and direct human impacts negates the protection they provide during extreme events and reduces their adaptive capacity, with significant environmental, social and economic consequences for coastal communities.

## 2. Mangrove Vulnerability to Climate Change

It has been substantially demonstrated that mangroves are sensitive to projected climate change [24]. the primary impact likely to be rising sea level, affecting inundation period, productivity and sediment budgets to cause dieback from the seaward edge and migration landward, subject to topography, and human modifications [25,26,27,28,29]. Climate warming is likely to have little negative impact, even increasing mangrove productivity and biodiversity at higher latitudes [24,28,30,31,32,33].

Rainfall changes are of greater significance to mangroves, particularly reduced rainfall, with drier coastal areas showing lower tree stature and biodiversity relative to humid coastlines [34]. Reduced rainfall may change sediment inputs and salinity to affect productivity [35,36,37]. However, the effects of relative sea level rise are the primary climate change impact of concern, giving a range of severely detrimental effects on mangroves.

Sea-level rise was globally projected to be 0.18–0.59 m by 2099 (1.5–9.7 mm a^−1^) [38] and subsequent assessments consider this to be underestimated; it could be 1 m or more [39,40,41]. Sedimentation in mangroves allows the mangrove substrate to “keep up” with sea level rise, and so reduce impacts of increased inundation stress, as a natural adaptation process in mangrove systems. Different contributions to mangrove sediment accretion are organic detritus from the mangroves, mineral sediment from river discharge, and soil volume change/compaction [27,42,43]. For a vulnerability assessment the net vertical accretion that is the consequence of all of these is a sensitivity factor, referring to the characteristics of a system in relation to tolerance to change [44].

Tropical Africa is predicted to experience among the most dire consequences of global climate change [45], particularly low lying deltas in countries with low GDP limiting adaptive capacity. The IPCC synthesis report stated with high confidence that by the 2080’s, many millions more people will experience floods every year due to sea level rise [45], and Cameroon has been shown to have particular vulnerability to sea level rise [46]. On these coastlines sedimentary sheltered areas are dominated by mangroves which provide coastal accretion, stability and protection values, as well as a food resource for local communities [16,47]. Direct human pressure has resulted in significant mangrove losses in the last few decades [16,20,48], to which sea-level rise impacts will add further pressure.

This study uses interdisciplinary approaches to assess the vulnerability of a key mangrove area of Cameroon, Central Africa to the impacts of sea-level rise. This site was selected owing to well documented heavy human usage of the resource adding pressures to the ecosystem [47,48,49], combined with having a microtidal range rendering the intertidal habitat more subject to disruption by sea level rise [50]. This is because of the differing degrees of habitat change and disruption associated with different tidal ranges. For example 70 cm of sea level rise implies a 100% change in a mangrove habitat with a 1.4 m tidal range but only a 20% shift in one with a 7 m range.

Mangrove climate change vulnerability assessment methodology has eight components: forest assessment of mangroves, recent spatial changes of mangroves, ground elevations in and behind mangroves, relative sea level trends, sedimentation rates under mangroves, adjacent ecosystem resilience, climate (rainfall) modeling and compilation of local community knowledge [51]. This assessment from Cameroon investigates the key parameters of vulnerability related to low tidal range sites, of spatial change, elevations and sedimentation rates.

## 3. Environmental Setting

Cameroon has a total mangrove area of 1,957 km^2^, and with 6 true native mangrove species present. The dominant species is *Rhizophora racemosa*, accounting for over 90% of mangrove forest including seaward zones, and other species present are *R. mangle* and *R. harrisonii*, *Avicennia germinans* (Acanthaceae), and towards land *Laguncularia racemosa* and *Conocarpus erectus* (both Combretaceae) [52,53]. All of these species are of the Western mangrove species group, found in America and West Africa [54]. Basal areas in the Douala-Edea mangrove reserve are high at 31 m^2^/ha in unexploited forest, reducing to 10.3 m^2^/ha under moderate exploitation and 3.9 m^2^/ha under heavy exploitation [51,53]. Undergrowth in upper zones can include the pan tropical *Acrostichum aureum* (Pteridaceae) where the canopy is disturbed. *Nypa fruticans* was introduced in 1910 from SE Asia, and today remains occasional.

Mangrove associate species occurring on landward margins include *Annona glaba* (Annonaceae), *Cocos nucifera* (Arenaceae), *Guiborutia demensei* (Caesalpiniaceae), *Achornea cordifolia* (Euphorbiaceae), *Dalbergia ecastaphylum* and *Drepanocarpus lunatus* (both Fabaceae), *Athocleista vogeli* (Loganiaceae), *Pandanus candelabrum* (Pandanaceae), *Hibiscus tilaceus* (Malvaceae), and *Bambusa vulgaus*, *Paspalum vaginatum*, and *Sesuvium portulacastrum* (all Poaceae) [53].

**Figure 1:**
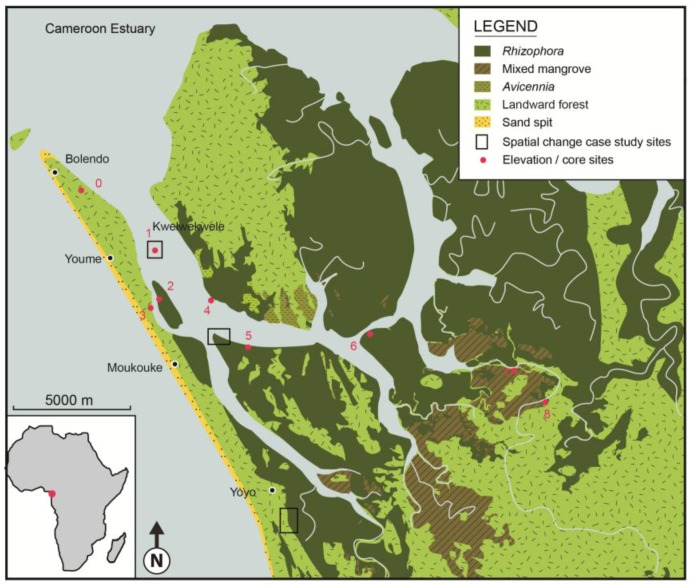
Cameroon Estuary southern mangrove area showing mangrove species zones and locations of spatial change case study sites.

The Cameroon Estuary (Figure 1) is one of three significant mangrove areas in Cameroon, located south of the horst of Mount Cameroon (4,095 m) in the central section of the coast where a number of riversdischarge [47,48,49]. Douala, the largest city in Cameroon borders the mangroves to the East and the mangroves are a significant resource to local communities through fishing, hunting and especially logging for fuelwood and charcoal [47,48,49]. On the southern shore of the Cameroon Estuary is the Douala-Edea Wildlife Reserve, with a total area of 1,600 km^2^ [53].

Coastal littoral drift carries sediments from the Sanaga main river mouth located south of Figure 1, creating a 15 km long coastal sand spit that encloses the Cameroon estuary mangroves from wave action, and along which a number of villages are located that are dependent on mangrove resources [47]. The climate is equatorial with generally warm temperatures with a mean monthly average of 26.5 °C and abundant rainfall of 3,000–4,000 mm. Cameroon has a semi-diurnal micro-tidal regime [55,56]. Northern summer SW monsoonal winds tend to sweep sand into the Cameroon estuary mouth, where sand banks are evident on charts [56], and sand deposits occur on the northern edge of the mangrove margins (Figure 1), these are utilized as village sites and largely vegetated by *Cocos nucifera*.

## 4. Material and Methods

Using spatial techniques combined with ground truthing we mapped the current distribution of mangrove zones, and compared with historical spatial records to show change over the last few decades. We used low technology techniques to establish the tidal range and relative elevation of the mapped mangrove area. Stratigraphic coring and paleobiological reconstruction was used to show the longer term biological history of mangroves and net sedimentation rate, and oral history surveys of local communities were used to investigate evidence of recent change and identify possible causes.

To map recent and historical mangrove area coverage, satellite images for the Cameroon estuary mangrove area were acquired through the USGS Landsat image archives. Landsat imagery was used for this study because Landsat has the longest image archive, dating back to 1973. However, due to heavy cloud cover, which is common in such equatorial latitudes, the oldest cloud-free image of this area was from 1975. Table 1 gives information about the images used for analysis, complemented by ground-truth data.

GIS analysis firstly involved geometric correction of distortion in images using 67 ground control points, and resized to have the same spatial resolution during sub-setting of the image scenes, by use of Environment for Visualizing Images (ENVI) software image processing tools. Then digital shoreline mapping and analysis was performed following procedures first developed by [57]. Analysis of individual images was then undertaken and results compared through an overlay methodology using GIS.

**Table 1:** Characteristics of satellite images used in the Cameroon spatial change analysis.

Available image date	Image	Time interval (years)	Cumulative time relative to base (years)	Clear status
1975	Landsat MSS	base	base	Very clear
1986	LandsatTM	11	11	clear
2000	LandsatETM	14	25	Partially clouded
2007	LandsatETM+	7	32	Clear but with gaps due to sensor’s scan line corrector mechanism failure
2010		3	35	Ground truthing

The US Geological Survey’s Digital shoreline analysis system (DSAS) was then undertaken [58], using an application that works within ArcGIS 9.x software. The DSAS extension was designed to aid in historic shoreline change analysis [58] to compute rate-of-change statistics determined by fitting a least squares regression to a time series of shoreline positions to estimate rates of change of mangrove seaward edges and landward margins [59]. On the main body of the mangroves, linear regression rate of change (LRR) statistics were used to analyze 246 transect measurements perpendicular to the mangrove edge over the period 1975–2007. These locations were chosen because they encompass the seaward edges of the mangrove areas shown in Figure 1.

The DSAS approach established a baseline from which the different time series of mangrove margins were measured, using a 200 m buffer offset of the 2000 Landsat ETM+ image edge. The offset was converted into a polyline, which was split and the onshore or offshore segment was used as the baseline for some areas while the other corresponding segment was deleted. The mangroves seaward edges for the 1975 Landsat MSS, 1986 Landsat TM, 2000 Landsat ETM+ and 2007 Landsat ETM+ were generated through digitalization of the respective mangroves edges at a scale of 1:10,000 from the images. These years are evenly spaced through time so avoiding the main problem that occurs with linear regression that one early date and a cluster of recent dates can skew results [59]. The baseline and respective mangroves edges cardinalities were defined using transects spaced at 300 m cast perpendicular to the baseline.

Field ground truthing was carried out by boat, with a total of 67 GPS waypoints collected in the field with attribute information ranging from mangrove species type, location of specific features such as the seaward edge and landward margin of areas of the mangroves, shores of the offshore Kwelekwele Island, villages in and around the mangroves area, monitoring sites used for water level measurement, and the stratigraphy core site. These GPS points were entered in a database (Microsoft Access) and imported in to ArcGIS 9.3 for analysis.

To establish the longer term biological history of the site, a stratigraphic core was sampled from the seaward edge of mangroves, located 20 m inside the northern seaward edge of the mangrove swamp at Moukouke Island (site 2 in Figure 1), in 25–30 m tall undisturbed *Rhizophora racemosa* forest. The canopy was open with numerous young seedlings as undergrowth. This 2 m core was recovered using a Hiller corer of 28 mm internal diameter, for ease of penetration through mangrove roots and sand. Contamination was prevented by washing outside of the corer before opening, and dismantling and washing it before recovering the next lower section. Stratigraphy was described and sub-sampled at 10 cm intervals, with color of stratigraphic units was determined by comparison with Munsell Soil Charts, and texture determined by feel analysis [60].

The different contributions to mangrove sediment accretion, such as organic detritus from the mangroves, mineral sediment from river discharge and soil volume change or compaction can be concurrently measured using surface elevation tables [27,42,43]. Radiocarbon dating or lead-210 dating the last few decades [25] provides net accretion rates that represent the average for the entire record and do not include variation within different time periods [61]. Modern measurements of surface elevation change may overestimate it due to the short term record, while radiocarbon dating of cores may underestimate the ability of a mangrove system to build vertically [61], so providing a precautionary approach to the vulnerability assessment. To determine the net sedimentation rate, the base of organic strata was sampled for radiocarbon dating, and age determination was carried out by accelerator mass spectrometry, with acid wash pretreatments and δ13C determination. Calibration to conventional years was carried out using the Pretoria Calibration procedure [62].

Pollen analysis was carried out using standard techniques [63,64] modified for resistant mangrove sediments [65] to concentrate fossil pollen. To each sample a known number of exotic *Lycopodium* was added, to allow the determination of pollen concentration per sediment volume. Broadly negative relationships between pollen concentrations and sedimentation rates are expected in allochthonous environments where high sediment input dilutes the pollen concentration [66]. Pollen were identified by comparison with a reference collection and published descriptions [67]. Other palynomorphs such as fungal spores, microforaminifera, dinoflagellates and chlorophyllaceae were excluded from the count. Results were transferred into pollen diagrams using TILIA and TILIAGRAPH [68], showing the relative representation of each taxon as a percentage of the total pollen sum which includes mangrove taxa, non-mangrove trees and shrubs, ferns, herbs and aquatics. The remainder of each sample was analysed for percent organic matter content by loss-on-ignition at 550 °C for 4 hours [69], where higher organic content is indicative of mangrove strata relative to offshore deposits being more inorganic [70,71].

Relative topographic elevations of the seaward edge, mangrove zone margins, the landward edge of mangrove and the core site were determined by simultaneous measurement of water height at each location and also at a reference station [51,53], a technique refined from the high tide mark topographic technique of [72]. Measurement of water surface slopes within tidal creeks [73,74,75], all found that during rising tide the water surface slope is relatively level, thus comparison of its height above the mud surface can indicate relative elevation.

Social surveys in villages close to the mangrove area (Figure 1) were conducted using structured interviews, with the objective to interview senior members of the community regarding changes they had observed in the mangrove area over time. The target group was the aged population with past experiences of the region, and this proved to be a rarer age group. In total 5 persons were interviewed with ages ranging from 45 to 65 years old.

## 5. Results

Analysis of the most recent 2007 satellite image combined with ground truth observations and forest composition assessments [53] resulted in the map of mangrove zones of the Douala-Edea area shown in Figure 1. Analysis of spatial change 1975–2007 showed there has been an overall decline of 5% in mangrove area since 1975, and most of this occurred 1975–1986, with a slight recovery 1986–2000, followed by a further decline 2000–2007 (Table 2). During this time period of 1986–2007 the area used for agriculture remained stable, while the area of human settlements increased.

**Table 2:** Cameroon estuary spatial change in area of vegetation and landuse cover (hectares).

Year	1975	1986	2000	2007
Mangroves	18,258	17,190	17,580	17,279
Settlements	176	176	182	183
Agriculture	592	583	583	583
Mudflat/sand	613	308	303	265
Lowland Forest	21,662	20,504	21,927	22,183

*Rhizophora* was confirmed to be the most prevalent mangrove community, with small areas of mixed forest towards landward margins and small areas of *Avicennia*. The sand spit enclosing the mangrove area from the ocean was shown by GIS analysis to have prograded towards the north by about 1.1 km between 1975–2007, with most of this spit growth occurring in the most recent period 2000–2007.

**Figure 2:**
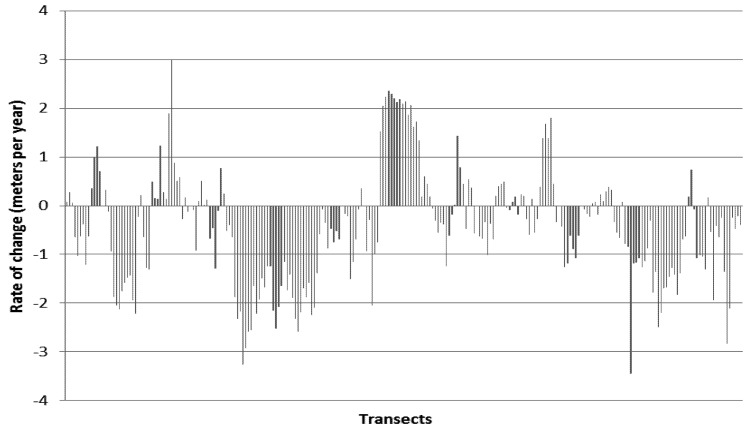
Rate of change results from 246 transects across the seaward edge of mangroves, Cameroon Estuary, 1975–2007. A negative rate of change shows mangrove retreat, while a positive rate of change shows mangrove advance or progradation.

The seaward edge of mangroves was analyzed in detail owing to its sensitivity to sea level rise impacts. On the seaward shoreline main body of the mangroves (excluding offshore islands), linear regression rate of change (LRR) statistics used to analyze 246 transect measurements perpendicular to the mangrove edge over the period 1975–2007. Results found that 79 showed a positive LRR (rate of mangrove movement seawards) with a maximum of 3.0 m a^−1^, mean 0.79 m a^−1^ (SD = 0.77), while 164 showed a negative LRR (rate of mangrove retreat landwards) of maximum of 3.4 m a^−1^, mean of 1.1 m a^−1^ (SD = 0.76), and 3 showed no rate of change (Figure 2).

**Figure 3:**
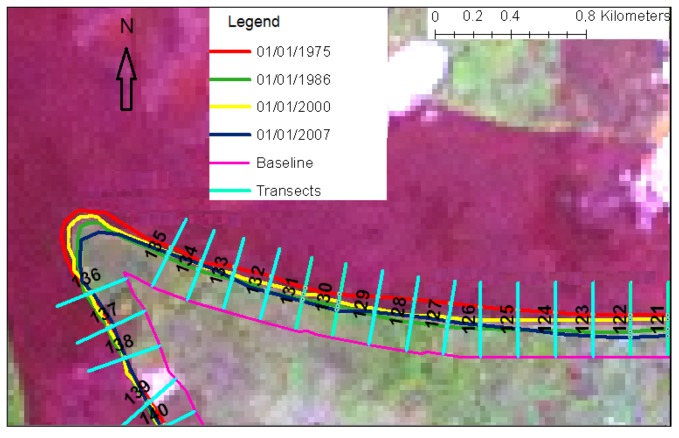
Seaward edge of mangroves showing change 1975–2007, demonstrating the DSAS technique of measuring change against a measurement baseline, by use of regular perpendicular sample transects.

These results are further illustrated in two seaward edge case studies located in Figure 1. Figure 3 shows the seaward edge of part of the main body of mangrove forest, closest to the ocean entrance to the Cameroon estuary as shown by the central case study box in Figure 1. This edge of the substantial mangrove forest area showed loss or extension of +/−30 m at most (Figure 3), with the majority showing slight retreat. This was found to be typical of results from elsewhere along the seaward edge of larger areas of mangrove.

**Figure 4:**
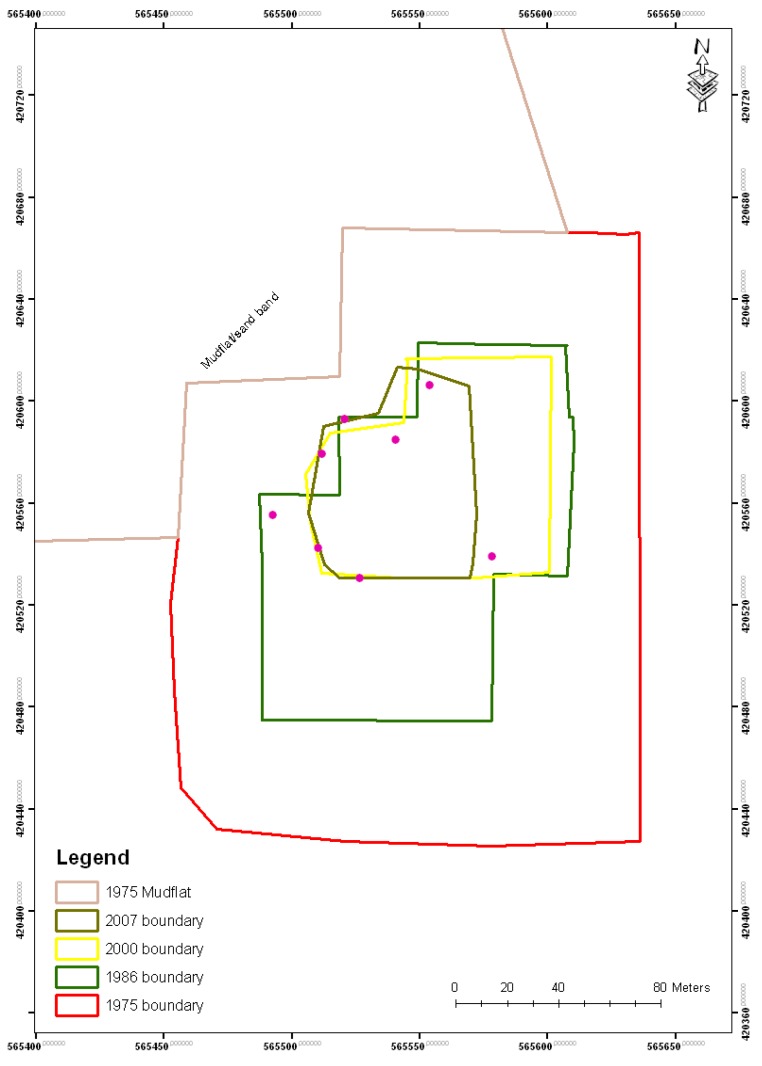
Seaward edge retreat of Kwelekwele Island from 1975 to 2007.

**Figure 5:**
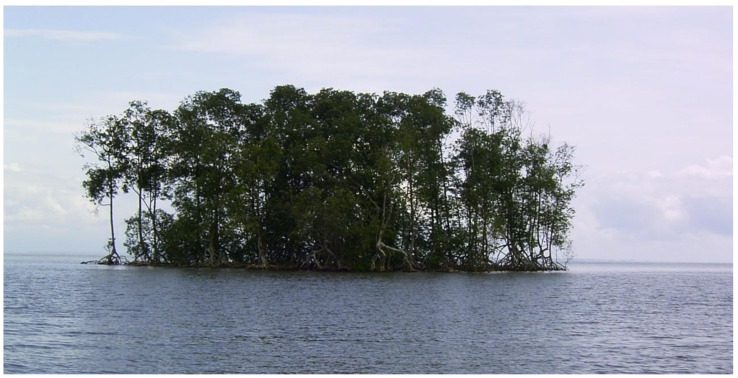
Kwelekwele Island in June 2009, showing evidence of forest retreat with open trees at the island edge and lack of canopy cover descending to near water level that is normal for seaward edge mangroves.

By contrast, the offshore mangrove island Kwelekwele Island (the northern case study box in Figure 1) was shown to have significantly reduced in area over the time period (Figure 4). This mangrove island was observed early in the study to have evidence of forest retreats with open tree trunks visible at the island edge (Figure 5), while it is normal for the seaward edge of mangroves to have dense canopy cover descending to near water level as the mangrove foliage takes advantage of light availability. The spatial change discovered at Kwelekwele Island was quantified using GIS to give the area change results in Table 3, showing that by 2007 this offshore mangrove island had declined to 11% of the area that it covered in 1975.

**Table 3:** Kwelekwele island mangrove area change 1975 to 2010.

Year	Island area (m^2^)
1975	39,200
1986	17,300
2000	6,900
2007	4,400
2010	4,300

**Figure 6:**
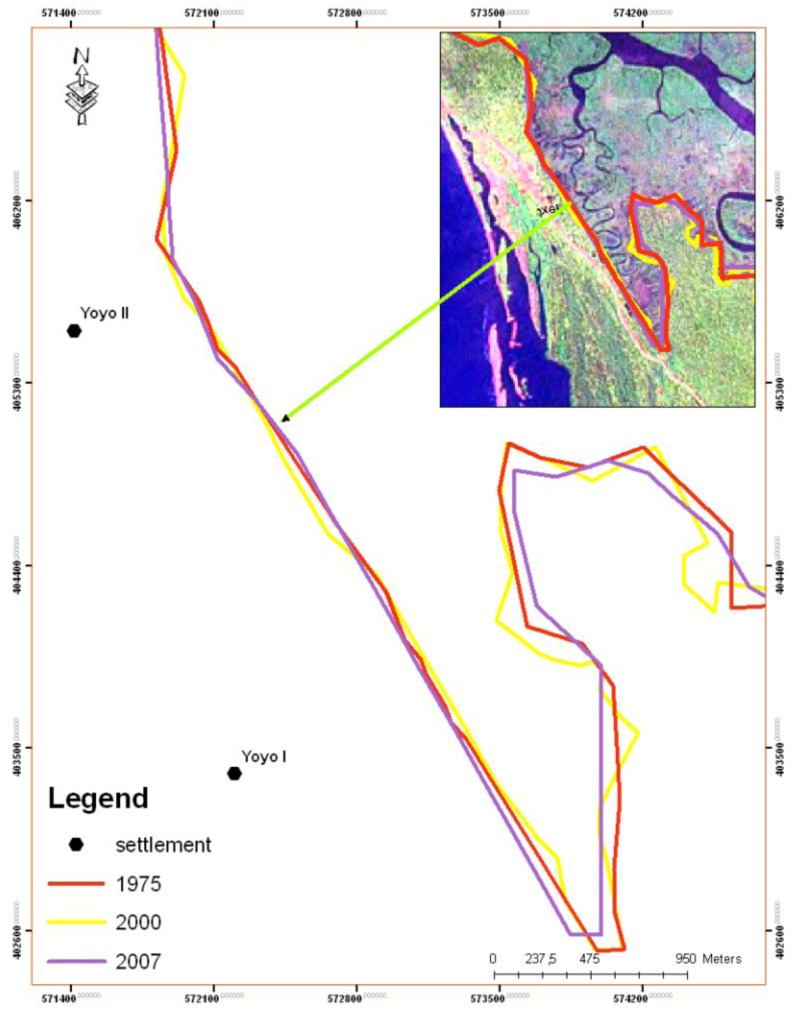
Mangrove landward margin change; near to Yoyo I and II villages segments, Cameroon estuary, Cameroon.

Comparison of satellite imagery 1975–2007 showed the landward edge of mangroves with lowland forest to show some changes, as demonstrated by a case study from near Yoyo village (the southern case study box on Figure 1) shown in Figure 6.

**Figure 7:**
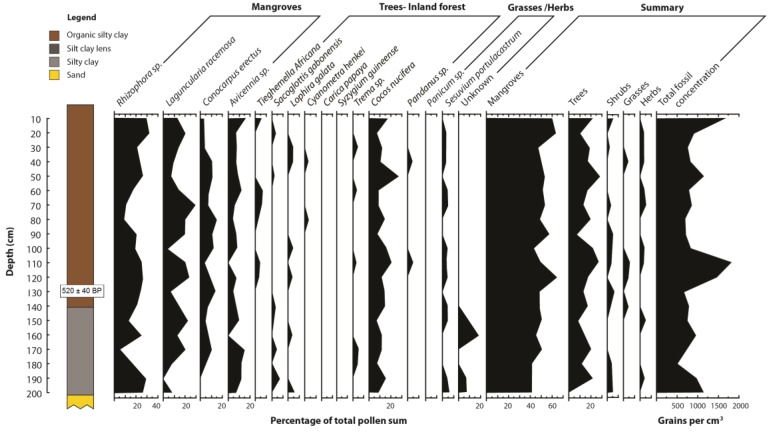
Stratigraphy and pollen results of the Moukouke Island core. Pollen is shown by percentage of pollen presence of the total pollen sum, and total fossil pollen concentration.

**Figure 8:**
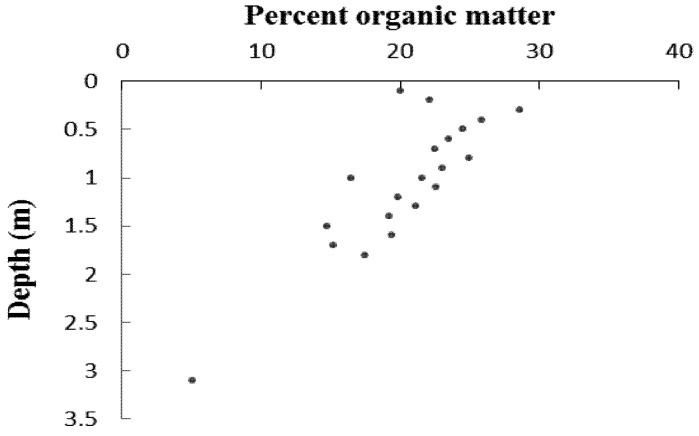
Percentage organic results from the Moukouke Island core.

Longer term results from stratigraphy of the Moukouke Island core taken at site 2 on Figure 1 showed a record of loose organic silty clay from the surface to 1.40 m (very dark grey 7.5YR 3/1) (Figure 7), becoming more compact with depth. Calcareous shell fragments increased in occurrence with depth. Below 1.40 m was consistent dark grey silty clay (7.5YR 4/1) to 2.00 m. There was increasing shelly sand content below 2.00 m, and the core ended in solid sand at 3.10 m. There was no macro-fossil evidence of mangrove presence below the organic silty clay upper layer, with stratigraphy below this shown to be inorganic. Percentage organic matter results (Figure 8), showed a consistent decline with depth in the stratigraphic core of organic fraction from 20%–30% near the surface to c. 15% below 1.50 m.

The AMS radiocarbon dating sample was taken from the base of the more organic surface unit of the core where the percentage organic matter was falling to below 20% (Figure 8). This result is shown in Table 4, along with the long-term net sedimentation rate calculated from the calibrated date and depth. The δ13C result is typical of mangrove strata [76].

**Table 4:** Radiocarbon dating results from Moukouke Island core, Cameroon estuary.

Sample	Depth (mm)	^δ13^C	^14^C age (year BP)	Calibrated years before present	Sedimentation rate (mm a ^−1^)
Beta-261195	1,300	−26.4	540 ±40	520 ± 40	2.5

The pollen diagram (Figure 7) of the stratigraphic core at Moukouke Island shows the relative representation of each mangrove taxon as well as the total mangrove pollen proportion of this pollen sum as a percentage of the total pollen sum which includes mangrove taxa, non-mangrove trees, shrubs, herbs, aquatics and ferns. In the 3.00 m core, mangrove pollen was dominant at 60% of the total sum 0–20 cm depth, falling to c. 40% lower in the core (Figure 6). *Rhizophora* was found at 30% of the pollen sum at the top of the core, falling to 10%–30% below 30 cm, combined with very low pollen concentrations of between 500–1,000 grains cm^−^^3^, with some outliers up to 1,500 grains cm^−^^3^, which is low for a mangrove forest of this species group [77]. *Laguncularia racemosa*, *Conocarpus erectus*, and *Avicennia marina* also occurred but at lower proportions (Figure 7). Throughout the core there was a significant presence of *Cocos nucifera* pollen, at 10%–20% of the pollen sum, there being significant *Cocos* plantations on the sand ridges adjacent to the core 1 site (Figure 1). Pollen of littoral shrubs such as *Hibiscus* and *Gardenia* were very occasional and were excluded from the pollen diagram.

Relative elevation results are show in Table 5, with the range of mangroves shown to be between 6.8 and 75.4 cm above the reference station, across a 69 cm elevation bracket. Elevations under mangroves on Kwelekwele Island (Figure 4 and Figure 5) are about 35 cm lower than seaward edge mangroves elsewhere in the estuary. *Rhizophora racemosa* showed the broadest elevational range of about 6–54 cm. *Laguncularia* and *Avicennia* were shown to have narrower elevation brackets of 20 cm or less.

In village interviews with senior citizens, most of those interviewed could remember that they either visited Kwelekwele Island (Figure 5) in the late 1970’s and early 1980’s or they lived there. They related that it was a major fishing market for the region. Then increased flooding that appeared to them as rising water caused constant destruction of buildings, and the inhabiting population began to reduce gradually, until there was a major inundation on the island in the mid-1980s which finally caused the remaining population to move out. The reason for the inundation was thought to be due to the vexation of the mermaid (a mythical sea creature believed to be in control of the sea and lands close to it). They did not think it is possible to reinstall the island.

**Table 5:** Relative elevation of mangrove substrate surface of the Doula-Edea mangrove area.

Station	Location	GPS Position	Mangrove zone	Elevation (cm)
0	Reference station Jonathan Creek	03° 48' 01.9" N 09° 34' 04.4" E	Seaward of mangroves	0
1	Kwelekwele Island	03° 48' 17.6" N 09° 35' 24.0" E	*Rhizophora racemosa*	6.8
2	Moukouke Island core site	03° 45' 54.9" N 09° 35' 40.4" E	*Rhizophora*	47.0
3	Seaward edge	03° 45' 27.5" N 09° 35' 37.6" E	*Rhizophora* and *Avicennia*	44.4
4	Seaward edge	03° 45' 35.1" N 09° 37' 09.0" E	*Avicennia*	21.3
5	Mid swamp	03° 44' 31.9" N 09° 37' 52.6" E	*R. mangle*	75.4
6	Mid swamp-Nkamba	03° 44' 46.7" N 09° 40' 41.42" E	*R. racemosa*	54.1
7	Landward edge	03° 43' 58.6" N 09° 44' 02.2" E	*Laguncularia* with some *Raphia* palms	73.1
8	Landward edge	03° 43' 05.6" N 09° 45' 09.0" E	Freshwater swamp	75.2

## 6. Discussion

Spatial analysis can identify and quantify mangrove retreat at the seaward edge, recruitment inland [78,79,80] or stability of mangrove distributions. Vulnerability is shown by change in mangrove area over time, caused by both human impacts and influences such as sea level rise. Lack of spatial change shows resilience of the mangrove system over time, which maintains older-growth trees and better reproductive success. Sea level rise impacts are shown by consistent mortality and retreat at the seaward edge and inland recruitment at the landward edge, while expansion of the seaward edge over time suggests either a strong sediment supply or sea level fall and is a sign of good mangrove resilience. Retreat of the seaward edge over time, if consistent along the coast, very likely shows vulnerability to sea level rise.

Spatial change analysis of mangroves of the Douala-Edea mangrove area 1975–2010 has shown that the area lost 979 hectares of mangroves 1975–2007 (Table 2), declining to 94% of its previous area. The area of lowland forest increased, partly owing to the extension of the sand spit to the west of the area, and the area of human settlements increased. The majority of the seaward edge of the main body of mangroves retreated between 1975–2007, with 67% of transects showing a negative rate of change, of a mean rate of −1.06 m a^−1^, while only 31% showed a positive rate of change of mean +0.79 m a^−1^.

The offshore mangrove island Kwelekwele however suffered a consistent loss over the period, losing a total of 3.49 hectares (Table 3) or declining to only 11% of the area that it had in 1975. Through community surveys this was found to have been linked with human settlement on the island, which likely caused increased pressure on the forest resources for fuelwood, combined with substrate disturbance and disaggregation that would have led to erosion during high tides, leading to inundation stress to the remaining trees. Greatest rates of island loss were found to have occurred 1975–1986 (Table 3, Figure 4), when according to interviews the island was inhabited. 

Longer term history as evident from the core taken from the mangrove seaward margin showed a stratigraphy in the Cameroon estuarine mangrove area of lower levels of inorganic sand at 2.0–3.5 m below the mangrove surface (Figure 7), fining upwards to inorganic silty clay to 2.0–1.4 m below the mangrove surface, with shallow levels of more organic silty clay above this from 1.4 m below the surface. The sand to silt fining upwards sequence demonstrates an environment of decreasing wave and/ or current energy, followed more recently by colonization by mangroves as indicated by the upper organic content to the silty clay. Percent organic matter levels are slightly higher in this surface unit, but are overall very low, indicative of significant allochthonous inorganic sediment input. Pollen concentrations are also very low at c. 1,000 grains cm^−^^3^ (Figure 7) compared with study sites also having the Atlantic mangrove species group [66,76,77], further indicative of inorganic sediment dilution of pollen deposition. 

In the surface organic mangrove unit, the net sedimentation rate was shown to be 2.5 mm a^−1^ (Table 4). This is similar to elevation change rates shown by surface elevation tables and marker horizons for a fringe and basin mangroves of the same species in Belize and Florida [42]. From stratigraphy in West Papua net mangrove sedimentation rates were found of 0.6–1.5 mm a^−1^ [81]. If accretion is at the same rate as relative sea level rise, then tidal inundation frequencies are maintained and mangrove vulnerability to rising sea level is much reduced.

These rates are however low relative to sea level rise projections; rates projected by the IPCC 4th Assessment are of 1.5–9.7 mm a^−1^ [38]. Furthermore, global mean sea level has already been rising, at a global average rate of sea level rise of 1.8 ± 0.5 mm a^−1^ between 1961 and 2003. Estimates of sea level change for the coast of Cameroon were calculated using satellite data and the Takadori, Ghana tide gauge data 1930–1965 to give a corrected rate of 1.8–2.2 mm a^−1^ for the period 1948–2003 [46]. These rates of recent sea level rise are close to the net mangrove accretion rate from core 1 (Table 4), and would have contributed to the increased inundation observed by residents of the low-lying Kwelekwele island. 

Kwelekwele Island mangrove substrate surfaces were shown to be about 35 cm lower than seaward edge mangroves elsewhere in the estuary (Figure 4 and Table 5), contributing to its vulnerability. Community knowledge of older residents of nearby villages confirmed this loss of island area and related increased inundation at a time when sea level rise is estimated to be 1.8 to 2.2 mm a^−1^ [46]. This case study demonstrates how offshore islands at the seaward edge are especially vulnerable to rising sea level owing to their lower elevation, particularly when combined with human disturbance. The lower elevation increases inundation levels to disadvantage mangrove productivity, while the offshore location (Figure 1) reduces sediment supply as it is further from riverine sources. 

The pollen diagram results showed higher proportions of mangrove pollen of 60% at the surface under well-established tall *Rhizophora* stands down to 30 cm depth (Figure 7). Below 40 cm mangrove pollen declines to around 40%, which combined with very low concentrations and shell presence probably represents offshore tidal flats. This seaward edge core was at the lower elevational range of mangrove tolerance (Table 5), hence the 30 cm depth of organic mangrove stratigraphy and the low mangrove pollen proportions lower in the core indicates that recent sea-levels were 10–20 cm lower than present, which corresponds with global trends in the last 100 years [82].

The range of mangrove substrate elevations (Table 5) shows about a 70 cm elevation bracket in a tidal range of 1.2 m, and it is normal for mangroves to occupy the upper half of the tidal range [83]. *Rhizophora racemosa* has the broadest elevational range of about 48 cm, which would make it the most resilient species to sea level rise of all present, in tolerating a changing sea level [50,51]. *Laguncularia* in particular, and *Avicennia* both have narrower elevation brackets (Table 5), making them less able to tolerate a rising sea level.

The Cameroon Estuary mangroves of Cameroon show resilience in the area of mangroves reducing by only 5% 1975–2007 but this study also demonstrates predominant seaward edge retreat, and some additional inherent vulnerability due to the low tidal range of the area. Vulnerability can be reduced by addressing the non-climate stressors on the mangrove area, particularly those resulting from human impacts, and by fostering management actions that enhance sedimentation rates. Priorities for adaptation planning in mangrove areas that are located in such low tidal range regions are to plan inland migration areas and strategic protected areas for mangroves, and to undertake management activities that enhance accretion within the mangroves.

Protected areas support key centers of biodiversity and provide refuges for wildlife, and mangroves are frequently underrepresented ecosystem types in marine protected areas [84]. Mangrove protected areas that are strategic choices in light of climate change are those that have a good sediment supply and high species diversity, as both of these factors enhance resilience.

Long-term planning of strategic protected areas is improved if these areas have designated inland migration areas defined by elevation for sea level rise of up to 1 m and more. Also, larger reserves better ensure representation of all mangrove community types to spread risk and increase chances for mangrove ecosystems to adapt to climate change and other stresses [28,85]. Areas that have a microtidal range have unique mangrove settings that are important to protect; and, although they have higher exposure to sea level rise, their vulnerability can be reduced by adaptation actions that reduce other stresses.

The habitat stability of mangroves depends on the maintenance of soil elevation relative to sea level, which, in the case of sea level rise, requires surface accretion. This allows mangroves to naturally adapt to rising sea level and can be facilitated by managers. Reduction in sediment supply at the coastline can result from increased human population at the coast and associated development, such as jetties that starve down-drift sections of sediment supply [86]. Dam construction on rivers reduces the volume of water and riverine sediment supply to the sea and coastal mangroves [87], which can lead to a sediment supply deficit which contributes to the increased vulnerability of mangrove areas to rising sea level, as resilience depends on sediment supply. Management actions to enhance sedimentation in mangroves therefore need to include coastal planners, infrastructure managers and river management agencies to build in design components that ensure continued sediment supply to the mangrove areas.

Root mat growth has been found to be a major contributor to surface elevation gain and is enhanced when mangroves are more productive [61]. Root mat growth has been shown to be higher under dense, healthy mangrove forests and lower under dwarf or scrub mangroves [42]. Enhancement of the productivity of mangroves leads to marsh elevation gain [88]. Hence improving the condition of mangroves also promotes accretion in those mangroves, which can be done by reducing human pressure, to enhance root mat growth and so reduce vulnerability to rising sea level.

## 7. Conclusions

This study demonstrates how an interdisciplinary combination of approaches can quantitatively assess the vulnerability of a sensitive ecosystem such as mangroves to climate change influences. Spatial change analysis of the Doula-Edea mangrove area has shown predominant seaward edge retreat in recent decades, and marginal mangroves subject to increased human pressure and ocean influences have shown decimation. Long term contexts from stratigraphic pollen analysis showed that the net sedimentation rate is close to current rates of sea-level rise, but the mangrove area has in the past shown resilience and progradation owing to sediment supply. Low technology measurement of relative elevation of the large mangrove area showed that it is located in only a 70 cm elevation bracket, hence its vulnerability to projected sea level rise of equal to above this range is high. 

Stratigraphic and pollen analysis results of this study indicate that sea-level has been stable on the central Cameroon coastline until recent global sea level rise caused by industrial greenhouse emissions, and with fairly rapid inorganic river-dominated sedimentation rates, mangroves have been prograding seawards. This is shown by higher levels of mangrove pollen in stratigraphy only at shallow depths where mangroves currently occur, and is supported by low pollen concentrations and low organic fraction in stratigraphy. 

In the last few decades global mean sea level has been rising [82], at an average rate of sea level rise of 1.8 ± 0.5 mm a^–1^ between 1961 and 2003, and stratigraphy shows that mangroves have recently been present up to 30 cm below current seaward elevations supporting that such trends have also been the case for this area. Mangrove loss has been indicated by GIS spatial evidence of predominant retreat of the seaward edge and decimation of an island offshore, an event supported by oral history evidence.

Vulnerability can be reduced by reduction of other impacts on mangrove productivity, particularly by ceasing any fuelwood harvesting from seaward edge locations, and reducing this elsewhere as propagules are less likely to establish on cleared areas [89]. Adaptive capacity can be increased by enhancement of sedimentation through restricting any further dam development on rivers, restricting coastal constructions that block sediment supply, and enhancing mangrove ecosystem health. 
